# Case of Splenic Abscess

**Published:** 1873-12

**Authors:** J. Knowles

**Affiliations:** Villisca, Iowa


BUFFALO

### Medical and Surgical Journal.

VOL XIII. DECEMBER, 1873. No. 5.

### Original Communications.

:o:

ART. I.—*Case of Splenic Abscess.* Reported by J. KNOWLES, M. D., Villisca, Iowa.

I was called on the evening of August 7, 1873, to see a young man, aged twenty, by occupation a farmer, who was suffering with remittent fever. On the morning of that day he felt somewhat indisposed, but still was feeling able to work; but about noon he was taken with a chill followed by fever. I saw him about 10 o'clock P. M. I found his pulse about 95, tongue coated, face flushed, and eyes suffused. He complained of pain in the right and left hypochondriac regions, also of severe headache and soreness and aching of the back and extremities; no vomiting, and no tendency that way; bowels had not moved for thirty-six or forty-eight hours. I prescribed Compound Cathartic Pills, U. S. P., three to be taken immediately; and in case the bowels did not move in three or four hours, to repeat the dose. After the bowels had moved he was to take quinine, grs. v., every four hours during such time as he had not much fever. During the time that the fever ran very high I ordered verat. virid. to be given.

I saw him again August 8th, 7 P. M. His bowels had moved freely after the second dose of pills. Quinine had been given from about 4 A. M. of that day, with the exception of one dose in the afternoon when the fever was highest. His pulse was 82. His tongue was looking better, and he was perspiring a little. I continued to prescribe the quinine with the addition of a little morphia sulphas to quiet the pain in the hypochondriac region, which still annoyed him some. Ordered warm fomentations over the seat of the pain, which, by the way, was ordered the day before.

August 10th, I saw him again. His pulse was 68 and natural; bowels had moved once during the day; his skin was moist, and he was free from pain; had eaten some light food during the day, and relished it. I put him on quinine and iron, in good doses, to be continued for several days, one dose every five to six hours. I then discharged the case on condition that he should let me know if he did not get right up. I also cautioned him against going out too soon, as the weather was very hot.

On the evening of August 20th I saw the case again with Dr. McNaughton. Our patient had been worse for several days, but had neglected to let me know until that time. He was at this time suffering intense pain in the left hypochondriac region; his tongue was not much coated; his pulse 80 per minute; bowels had not moved for thirty-six hours; no headache; skin dry and harsh. We now diagnosed the case one of Active Congestion of the Spleen. Upon inquiry we ascertained that the patient had done well for several days, and thinking it would do no harm had exposed himself for two or three hours to the sun while the thermometer ranged 98° in the shade. From that time until our visit he had suffered as above described. We gave him powders containing each morphia sulph., 1-6 to ¼; quinine, gr. iv. to v., every four hours; also Fl. Ext. Buchu, et. Spr. Nitre Dulc., equal parts, one teaspoonful every four hours alternately with the powders; also ordered a blister of Cerate Canthar. over the left hypochondriac region. From the 20th to the 26th he remained about the same. On the evening of the 30th we were called in haste to see him. We found him still complaining of the pain in his side, tongue dry, bowels constipated, pulse rapid, weak and intermitting. We ordered his bowels moved by enema; continued the morphia and quinine every four hours, with whisky in tablespoonful doses every hour; also repeated the blister.

September 6th we both saw him again, and came to the conclusion that we had better look out for Splenic Abscess. Our patient suffered so much that we were compelled to keep him under the influence of an opiate pretty much all the time. He was troubled with profuse perspiration, so that his clothing would sometimes be wringing wet. We still stuck to quinine; also put him on Tr. Ferri chlo.; whiskey every hour as before. We also bathed him with spirits and capsicum to prevent the excessive and exhausting perspiration. A few days later we discovered a slight bulging of the surface beneath the last rib, and about as far back as the middle of the latissimus dorsi muscle. We now made up our minds that our former opinion was correct, and that the thing to do was to husband the powers of life to the best of our ability. We kept him on milk, beef tea, and, in fact, anything we could induce him to eat that was easily digested and nourishing. We kept up our tonic and stimulant treatment. On the 15th I found him suffering from a pretty severe dysentery, but it was easily controlled by the use of starch and laudanum injections; also painted the tumor with tincture of iodine; tried to make use of a poultice, but as the patient had to lie all the time upon the left side they became so uncomfortable that their use had to be discontinued. On the 20th there was some superficial redness, and the swelling was increasing. On the 22d the redness and swelling was still more perceptible, with a small, soft spot about the centre.

On the 24th we decided to evacuate the pus. As the patient was very weak and nervous, Dr. McNaughton administered chloroform, when I proceeded to make a crescent-shaped incision through the integument and superficial tissues, then dissecting back a little, forced a scalpel into the abscess. At first the flow of pus was slow, but upon turning the patient on the left side it began to flow pretty freely. During the first twelve hours it was estimated that the discharge amounted to a quart of healthy pus. On the third day after opening the abscess it was necessary to break up some slight adhesions that had formed about the edge of the wound, after which the flow of pus continued for several weeks, gradually becoming less until the wound healed of its own accord. This evening (Nov. 15th) the patient was in our office looking well and hearty as ever in his life. One peculiar feature of the case was: That from the time of his relapse until after the opening of the abscess he was listless and careless and did not seem to care whether he lived or died, yet he was all the time conscious of his condition.

I report the case, as from consulting different authors I find Splenic Abcess to be one of no frequent occurrence, and for that reason may be of some interest.

To Dr. M. N. McNaughton I am under obligation for his friendly and timely counsel, and to his action in the matter much of the success is due.

